# Improved gene tree error correction in the presence of horizontal gene transfer

**DOI:** 10.1093/bioinformatics/btu806

**Published:** 2014-12-05

**Authors:** Mukul S. Bansal, Yi-Chieh Wu, Eric J. Alm, Manolis Kellis

**Affiliations:** ^1^Computer Science and Artificial Intelligence Laboratory, Massachusetts Institute of Technology, Cambridge, MA, USA, ^2^Department of Computer Science and Engineering, University of Connecticut, Storrs, CT, USA and ^3^Department of Biological Engineering, Massachusetts Institute of Technology, Cambridge and ^4^Broad Institute, Cambridge, MA, USA

## Abstract

**Motivation**: The accurate inference of gene trees is a necessary step in many evolutionary studies. Although the problem of accurate gene tree inference has received considerable attention, most existing methods are only applicable to gene families unaffected by horizontal gene transfer. As a result, the accurate inference of gene trees affected by horizontal gene transfer remains a largely unaddressed problem.

**Results**: In this study, we introduce a new and highly effective method for gene tree error correction in the presence of horizontal gene transfer. Our method efficiently models horizontal gene transfers, gene duplications and losses, and uses a statistical hypothesis testing framework [Shimodaira–Hasegawa (SH) test] to balance sequence likelihood with topological information from a known species tree. Using a thorough simulation study, we show that existing phylogenetic methods yield inaccurate gene trees when applied to horizontally transferred gene families and that our method dramatically improves gene tree accuracy. We apply our method to a dataset of 11 cyanobacterial species and demonstrate the large impact of gene tree accuracy on downstream evolutionary analyses.

**Availability and implementation**: An implementation of our method is available at http://compbio.mit.edu/treefix-dtl/

**Contact**: mukul@engr.uconn.edu or manoli@mit.edu

**Supplementary information**: Supplementary data are available at *Bioinformatics* online.

## 1 Introduction

Gene trees and species trees are the two fundamental types of phylogenetic trees. Although *species trees* represent the evolutionary history of a set of organisms (or taxa), *gene trees* represent the evolutionary history of a given gene family. Gene trees play a fundamental role in understanding gene family evolution (Thornton, 2004; Yang, 2007), and are used for inferring evolutionary events such as gene duplication and horizontal gene transfer (Choi *et al.*, 2012; Organ *et al.*, 2010; Zhaxybayeva, 2009), inferring orthologs, paralogs and xenologs (Koonin, 2005; Sennblad and Lagergren, 2009; Storm and Sonnhammer, 2002; Vilella *et al.*, 2009; Wapinski *et al.*, 2007), reconstructing ancestral gene content, and dating gene birth (Chen *et al.*, 2000; David and Alm, 2011; Ma *et al.*, 2008). These powerful applications of gene trees depend on the ability to infer them accurately. However, unlike species tree construction, which can benefit from the use of well-behaved orthologous gene families or multi-gene species tree reconstruction methods (Burleigh *et al.*, 2011; Delsuc *et al.*, 2005), accurate gene tree inference is confounded by the fact that most genes lack sufficient information to confidently support one gene tree topology over another. Indeed, previous studies (Li *et al.*, 2006; Rasmussen and Kellis, 2011) have shown that even the most accurate phylogeny reconstruction methods, such as neighbor joining (NJ) (Saitou and Nei, 1987) or maximum likelihood (ML) [e.g. RAxML (Stamatakis, 2006), PhyML (Guindon *et al.*, 2010)], that make use of sequence information alone, often produce erroneous estimates of the gene tree topology. To overcome this limitation, many *species tree aware* methods have been developed for gene tree inference. These methods make use of a known species tree and a reconciliation model that makes it possible to extract topological information from the species tree and use it to guide gene tree inference. Some of these methods, such as TreeBest (Vilella *et al.*, 2009), PrIME-GSR (DLRS) (Åkerborg *et al.*, 2009) and SPIMAP (Rasmussen and Kellis, 2011), reconstruct a gene tree directly from sequence data, but most, such as NOTUNG (Durand *et al.*, 2006), tt (Górecki and Eulenstein, 2011), TreeFix (Wu *et al.*, 2013), AnGST (David and Alm, 2011) and MowgliNNI (Nguyen *et al.*, 2012), take in previously reconstructed gene trees and *error-correct* them using the reconciliation model. In essence, these methods are based on the observation that any deviation of the gene tree from the species tree topology is due to the effect of evolutionary phenomena such as gene duplication, gene loss, horizontal gene transfer (or simply transfer for short) and incomplete lineage sorting (ILS).

In many eukaryotes (especially multi-cellular eukaryotes), duplication and loss play the dominant role in gene family evolution. This motivates the well-studied duplication-loss reconciliation model (Goodman *et al.*, 1979; Page, 1994) that accounts for any discordance between the gene tree and species tree by invoking duplication and loss events. Almost all existing species tree aware gene tree inference methods are based on the duplication-loss reconciliation model (Åkerborg *et al.*, 2009; Durand *et al.*, 2006; Górecki and Eulenstein, 2011; Rasmussen and Kellis, 2011; Vilella *et al.*, 2009; Wu *et al.*, 2013). However, gene families evolution is often more complex and is driven not just by duplication and loss but also by transfer events; this is common, for example, in microbial gene families. As a result, methods based on the duplication-loss model perform poorly and cannot be used for gene trees affected by transfer. Moreover, due to the complexity of the reconciliation model based on duplication, transfer and loss (DTL-reconciliation model), there has been little systematic development of species tree aware methods for handling gene families with transfer. Consequently, gene trees affected by transfer are difficult to infer accurately.

Two error-correction methods, AnGST (David and Alm, 2011) and MowgliNNI (Nguyen *et al.*, 2012), have recently been proposed for gene families with transfer. Both work by considering alternative gene tree topologies, reconciling them to the species tree and choosing the gene tree that yields the best (most parsimonious) reconciliation. AnGST accounts for uncertainty in gene tree topologies by taking as input, not a single gene tree, but a set of gene trees (e.g. bootstrap replicates) and creating a new gene tree that minimizes the reconciliation cost by piecing together clades from the set of input gene trees. MowgliNNI deals with topological uncertainty by considering alternative gene tree topologies obtained by performing nearest neighbor interchange (NNI) operations (see, e.g. Felsenstein, 2004) on branches of the original input gene tree that have lower support than some user-specified threshold and selecting a gene tree topology with minimum reconciliation cost as the inferred gene tree. Although AnGST and MowgliNNI are excellent first attempts, they have several limitations. First, they are both based on a simple parsimony criterion that ignores sequence support for the inferred gene trees, making them prone to over-fitting the gene tree to the species tree topology. Second, both consider only a limited search space, affecting their ability to recover the true gene tree topology. Third, MowgliNNI requires the input species trees be fully dated (i.e. all species tree nodes must be relatively ordered by time), which is notoriously difficult to do accurately (Rutschmann, 2006).

In this study, we introduce a principled error-correction based approach for species tree aware gene tree inference of gene families with transfer. Our method TreeFix-DTL attempts to find a tree that has the lowest reconciliation cost, under the DTL-reconciliation model, among all gene trees that have a likelihood that is ‘statistically equivalent’ to that of the ML tree. By balancing sequence likelihood with species divergence information from the species tree topology, TreeFix-DTL avoids the pitfalls of the existing approaches and finds more accurate gene trees. Similar ideas have been successfully used before in the context of gene tree error correction under the duplication-loss model (Wu *et al.*, 2013). The idea of statistical equivalence of gene tree likelihoods has been previously used to define ‘islands’ of trees (Salter, 2001; Sanderson *et al.*, 2011) and has also been used to study the congruence between species tree and gene tree topologies in bacteria (Lerat *et al.*, 2005).

To evaluate the performance of TreeFix-DTL, we develop a thorough simulation framework that accounts for variations in species tree sizes, rates of gene evolution, alignment lengths, rates of duplication, transfer, and loss, (DTL) and species tree errors. We use this framework to compare TreeFix-DTL to AnGST, MowgliNNI, RaxML, NOTUNG and TreeFix. RaxML is one of the most widely used phylogeny reconstruction programs, while NOTUNG and TreeFix are two well-known species tree aware gene tree error-correction methods based on the duplication-loss model. We observe that TreeFix-DTL outperforms all other methods and improves the accuracy of gene trees across all simulation parameters. Specifically, the contributions of this work are as follows:

1. **We demonstrate that methods that do not model transfer events do not work well for gene families with transfer.** We test the performance of NOTUNG and TreeFix on simulated gene families with transfer and observe that they show poor performance even on datasets with low rates of transfer.
2. **We introduce TreeFix-DTL, a statistically founded approach for species tree aware gene tree error** **correction in the presence of transfer.** We show that TreeFix-DTL outperforms all other methods at accurate gene tree inference and achieves a great improvement in accuracy compared with RAxML.
3. **We show that more accurate gene trees greatly improve the inference of evolutionary events.** We reconcile gene trees reconstructed using RAxML and those inferred using TreeFix-DTL, AnGST and MowgliNNI with their corresponding species trees and show that improved topological accuracy translates into a direct improvement in correctly inferring evolutionary events like duplications, transfers, and losses (DTLs).
4. **We show that TreeFix-DTL leads to an almost 10-fold reduction in the number of inferred transfer events on a dataset of 11 cyanobacterial species.** We apply TreeFix-DTL to a published dataset of 11 cyanobacterial species that has been previously analyzed for horizontal gene transfer and ILS, and demonstrate that, strikingly, most of the evolutionary inferences drawn using the original dataset change when TreeFix-DTL gene trees are used.

Throughout the rest of this study, all trees are assumed to be binary and, unless otherwise stated, gene trees are assumed to be unrooted and species trees rooted.

## 2 Materials and methods

In our experimental study, we applied the methods RAxML (Stamatakis, 2006), NOTUNG (Durand *et al.*, 2006), TreeFix (Wu *et al.*, 2013), MowgliNNI (Nguyen *et al.*, 2012), AnGST (David and Alm, 2011) and our new method TreeFix-DTL, to simulated datasets and evaluated the accuracy of the inferred gene trees. RAxML is a well-known method used for reconstructing a ML tree from sequence data and represents the baseline method for gene tree inference. NOTUNG and TreeFix are two of the best performing methods for species tree aware gene tree inference under the duplication-loss model (Wu *et al.*, 2013). MowgliNNI, AnGST and TreeFix-DTL are the three species tree aware methods available for gene tree error correction of transferred gene families.

Next, we introduce the DTL-reconciliation model, which is used by TreeFix-DTL as well as by AnGST and MowgliNNI, and then describe TreeFix-DTL. Finally, we describe the experimental setup and the synthetic and biological datasets used in the analysis.

### 2.1 DTL reconciliation

The DTL-reconciliation model allows for the reconciliation of a given rooted gene tree with a given rooted species tree by postulating DTL events. The problem of reconciling a gene tree with a species tree under the DTL-reconciliation model is referred to as the *DTL-reconciliation* problem, and has been extensively studied in the literature; see, e.g. Bansal *et al.* (2012) and the references therein. The DTL-reconciliation problem is typically solved in a parsimony framework, where costs are assigned to DTL events and the goal is to find reconciliation with minimum total cost. DTL-reconciliations can sometimes violate temporal constraints; i.e. the inferred transfers may induce contradictory constraints on the dates for the internal nodes of the species tree. Such paradoxical reconciliations are called *time* *inconsistent* (Doyon *et al.*, 2010).

Several formulations of the DTL-reconciliation problem have been studied in the literature: these include formulations where (i) the input species tree is undated and the goal is to find an optimal (minimum cost) time-consistent DTL-reconciliation, (ii) the input species tree is fully dated and the goal is to find an optimal time-consistent DTL-reconciliation and (iii) the input species tree is undated and the goal is to find an optimal (not necessarily time consistent) DTL-reconciliation. The first, undated formulation is known to be NP-hard (Hallett and Lagergren, 2001; Ovadia *et al.*, 2011; Tofigh *et al.*, 2011) and is therefore unlikely to be efficiently solvable. The second, fully dated formulation is known to be polynomially solvable (Libeskind-Hadas and Charleston, 2009) and the fastest known algorithms have a time complexity of $O(mn^{2})$ (Doyon *et al.*, 2010), where *m* and *n* denote the number of leaves in the gene tree and species tree, respectively. However, this formulation requires the use of a fully dated species tree, and accurately dating the internal nodes of a species tree is a notoriously difficult problem (Rutschmann, 2006). In contrast, the third formulation does not require a dated species tree and, since it relaxes the requirement for time-consistency, is known to be efficiently solvable in *O*(*mn*) time (Bansal *et al.*, 2012; Tofigh *et al.*, 2011). Thus, due to its wider applicability and efficient solvability, in this work, we will make use of formulation (iii) for the DTL-reconciliation problem, which assumes that the input species tree is undated and seeks an optimal (not necessarily time consistent) DTL-reconciliation. We note that MowgliNNI uses formulation (ii) of the problem and requires a fully dated species tree.

The DTL-reconciliation model can be easily extended to work with unrooted gene trees by considering all possible rootings of the gene tree and picking one that minimizes the reconciliation cost. Remarkably, the time complexity of formulation (iii) remains *O*(*mn*) even for unrooted gene trees (Bansal *et al.*, 2012).

### 2.2 TreeFix-DTL algorithm

TreeFix-DTL takes as input a single ML gene tree [obtained from programs such as RAxML (Stamatakis, 2006) or PhyML (Guindon *et al.*, 2010)], the gene sequence alignment on which the gene tree was built, and a rooted species tree. TreeFix-DTL then seeks a gene tree that has minimum reconciliation cost among all those gene trees that have likelihood statistically-equivalent to that of the ML gene tree. To implement this strategy, TreeFix-DTL performs local search starting with the given ML gene tree and successively finds statistically equivalent gene trees that have a lower reconciliation cost.

#### 2.2.1 The Shimodaira–Hasegawa test

TreeFix-DTL determines whether a tree is supported by the sequence data by computing the Shimodaira–Hasegawa (SH) test statistic (Shimodaira and Hasegawa, 1999). The test captures the observed likelihood difference between tree topologies chosen a priori, and then relies on hypothesis testing, for which the null hypothesis is that the trees are equally supported by the sequence data, and the alternative hypothesis is that the trees are not equally supported, to compute a *P*-value that represents the probability of obtaining a statistic as extreme as that observed assuming that the null hypothesis is true. At a user-defined significance level of *α* (default 0.05), corresponding to the probability of false rejection, we reject the null hypothesis and infer that the topologies are not equally supported if *P* < *α*, or we fail to reject the null hypothesis and say that the topologies are equally supported if *P* ≥ *α*. We tested the impact on accuracy of using different values for *α* (*α* = 0.1, 0.05 and 0.01) and observed that performance was robust to these changes (results not shown). Several implementations of the SH test exist, and, by default, TreeFix-DTL uses the SH test provided by the RAxML package (Stamatakis, 2006). In our simulation study (next section), we observed that only in 4.5% of the test cases did the true gene tree fail the SH test. This suggests that it is appropriate to limit the search space using the SH test.

#### 2.2.2 The role of DTL reconciliation

To determine an optimal gene tree among the set of proposed topologies supported by the sequence data, we incorporate species tree information by using the DTL-reconciliation framework Specifically, we use a parsimony based DTL-reconciliation framework (Bansal *et al.*, 2012) in which a lower total reconciliation cost implies a better fit with the species tree topology and consequently, a more optimal gene tree. Although evolutionary histories need not always be most parsimonious, our experiments suggest that parsimonious DTL-reconciliation is highly accurate, even for high rates of DTL (e.g. Fig. 2). We also point out that TreeFix-DTL only considers the cost of the reconciliation, not the actual reconciliation itself. In our simulation study, we observed that the true gene trees indeed tended to have the lowest reconciliation cost among all the gene trees considered during the heuristic search, suggesting that minimizing the reconciliation cost is an appropriate search strategy for finding more accurate gene trees. Only in 21.5% of cases did TreeFix-DTL output a gene tree with lower reconciliation cost than the true tree.

#### 2.2.3 Algorithmic details

TreeFix-DTL takes as input a ML gene tree *T*_ML_, a multiple sequence alignment *A*, and a rooted species tree *S* and outputs the best unrooted gene tree $T^{*}$ found during the search. To search the space of possible gene tree topologies, we employ a heuristic hill climbing strategy similar to the one used in TreeFix (Wu *et al.*, 2013), and based on NNI and subtree prune and regraft (SPR) operations (see, e.g. Felsenstein, 2004). During each search iteration, we start with the current optimal gene tree and propose a new tree by performing a random NNI or SPR rearrangement; this proposal is always accepted if it is statistically equivalent to the input gene tree and has lower reconciliation cost than the current optimal gene tree, and accepted with some predefined probability otherwise (to avoid getting caught in local minima). In case the search finds multiple gene trees with statistically equivalent likelihood and the same minimum reconciliation cost, it outputs the one that has the highest likelihood. By default, we perform 1000 search iterations which, in our study, worked well for a wide range of simulation parameters. Further algorithmic details appear in Supplementary Section S1.

Users can customize TreeFix-DTL by (i) specifying the significance level α∈[0,1] (set by default to 0.05) for likelihood equivalence, (ii) specifying alternative costs for DTL events to be used by DTL reconciliation (defaults set to 2, 3 and 1, respectively) and (iii) changing the thoroughness of the local search heuristic by specifying the number of search iterations (default 1000). The default values of these parameters have been set based on extensive experimental analyses. As we discussed above, performance is robust to the choice of *α* (we tried values 0.01, 0.05 and 0.1). For the event costs, our results show that the default costs work well for a wide range of datasets, and that, in general, performance is relatively robust to the exact event costs used. Finally, as discussed earlier, we chose a relatively high default of 1000 search iterations because it works well even for datasets with up to 200 taxa. We discuss the relationship between gene tree accuracy and the number of local search iterations in Supplementary Section S4.

### 2.3 Systematic evaluation using simulated datasets

#### 2.3.1 Basic experimental setup

To study the performance of the different methods, we created multiple simulated datasets, accounting for the key parameters likely to have an impact on gene tree accuracy; this includes rates of DTL; rates of sequence evolution; alignment length; and species tree size, where our choice of parameters are meant to simulate a wide variety of real gene families and are based on an analysis of a 4736 gene tree, 100 species dataset from David and Alm (2011), which consists of predominantly prokaryotic species sampled broadly from across the tree of life. Further details on the choice of parameters used appear in Supplementary Section S2.

Our basic simulation setup is as follows: we simulated 100 random species trees with 50 taxa, generated under a Yule process (Harding, 1971; Yule, 1925). For each of these 100 species trees, we then simulated gene trees under a probabilistic model of DTL (Tofigh, 2009; Tofigh *et al.*, 2011) with three different settings (low, medium and high) of duplication and transfer event rates. The chosen rates appear in Supplementary Table S1. The low-, medium- and high-DTL gene trees had, on average, 52.3, 70.4 and 91.3 leaf nodes, 1.2, 2.8 and 5.0 duplications, 2.2, 5.5 and 9.9 transfers and 2.1, 2.3 and 2.9 losses, respectively (Supplementary Table S2). For each of the three sets of 100 gene-tree/species-tree pairs, we simulated four different mutation rates by scaling the branch lengths of the gene trees so as to correspond to 1, 3, 5 and 10 mutations per site. Finally, we simulated amino acid sequences of length 173 and 333 down the gene trees in each set under the JTT model with gamma rate heterogeneity shape parameter 1 and 4 rate categories using the program Seq-Gen (Rambaut and Grassly, 1997). Thus, we created a total of 24 datasets, each with 100 gene-tree/species-tree pairs, and corresponding to a fixed rate of DTL (*low-*, *medium-* *and* *high-DTL*), a fixed mutation rate (*rate-1*, *rate-3*, *rate-5*, *rate-10*), and a fixed alignment length.

In addition to the basic simulation setup described earlier, we also simulated a number of datasets with larger species trees, shorter alignments, different rates of DTL, branch specific evolutionary rate variation and incorrect species trees.

#### 2.3.2 Choice of parameters for program runs

TreeFix-DTL, AnGST and MowgliNNI all require as input the costs for DTL events to be used with the DTL-reconciliation model. Based on existing literature (David and Alm, 2011), we set these costs to 2, 3 and 1, respectively for DTL. Note that we also ran TreeFix-DTL with event costs set to 1 each and observed that the ability to infer gene trees accurately were only slightly affected (Supplementary Fig. S3); this suggests robustness to the exact event costs used. Each of the species tree aware methods used in this study, TreeFix-DTL, AnGST, MowgliNNI, NOTUNG and TreeFix, require as input a gene tree reconstructed using sequence-only methods. We used the RAxML tree as the input gene tree for these methods. We used extremely thorough search settings when building the RAxML trees, picking the best tree out of 10 full RAxML searches, and with 100 rapid bootstraps (see Supplementary Section S3 for the exact command). Further details on the exact settings used for NOTUNG, TreeFix, MowgliNNI and AnGST appear in Supplementary Section S3.

## 3 Results

### 3.1 Gene tree accuracy

**Fig. 1.**
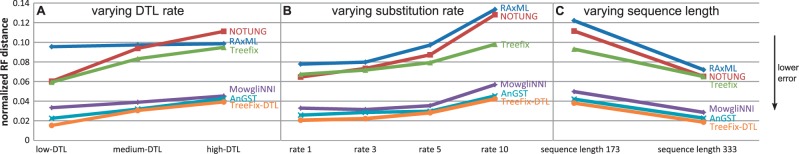
Error rates for different methods on simulated datasets. Error rates in terms of NRFD are shown for gene trees inferred using RAxML, NOTUNG, TreeFix, MowgliNNI, AnGST and TreeFix-DTL on the simulated 50-taxon datasets. Note that the lines are for visual guidance only. TreeFix-DTL produces the most accurate gene trees among all tested programs. (**A**) Results are shown for varying rates of DTL, averaged over all mutation rates and sequence lengths. (**B**) Results for varying mutation rates, averaged over all DTL rates and sequence lengths. (**C**) Results for varying sequence lengths, averaged over all DTL rates and mutation rates. Results for all 24 simulated datasets appear in Supplementary Figure S1

We evaluated the different methods on our simulated datasets (24 datasets, each with 100 gene-tree/species-tree pairs) and measured the accuracy of the inferred gene trees against the true gene tree topologies. We quantified the topological error in terms of the widely used Normalized Robinson-Foulds Distance (NRFD), which captures the fraction of splits in the two trees that are present in only one of the two trees. For example, an NRFD of 0.05 between two trees implies that 5% of the splits in the inferred tree are not present in the true tree and, consequently, that 5% of the splits from the true tree are not in the reconstructed tree. Thus, NRFD lies between 0 and 1, and the closer the value is to 0, the more accurate the gene tree. We found that TreeFix-DTL produced highly accurate gene trees over a range of evolutionary scenarios (Fig. 1; Table 1). Specifically, we observe the following:

**Fig. 2.**
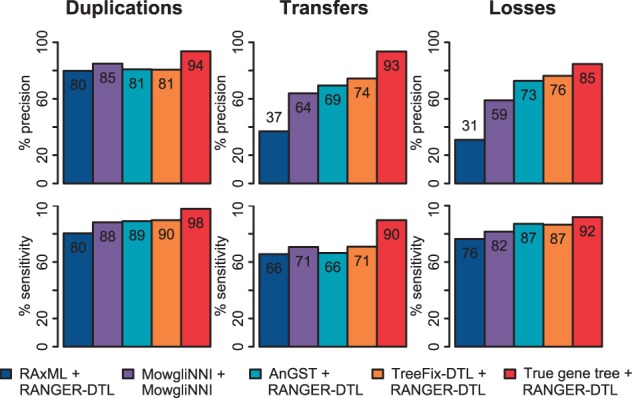
Accuracy of proposed DTL events. The precision and sensitivity of various phylogenetic programs at recovering DTL events, are averaged over all gene tree/species tree pairs from all 24 simulated datasets of 50 taxa. Although applying DTL-reconciliation to the true gene trees yields the highest precision and sensitivity, neither metric reaches 100%. Among all inferred gene trees, those from TreeFix-DTL yield the most accurate events

**Table 1.** Accuracy of the different methods at inferring gene trees

Method	NRFD	Perfect inference (%)
RAxML	0.097	3.04
NOTUNG	0.088	13.08
TreeFix	0.079	10.29
MowgliNNI	0.039	22.17
AnGST	0.032	29.08
TreeFix-DTL	0.028	38.21

Error rates are shown in terms of NRFD, and accuracy rates in terms of the percentage of inferred gene trees that have (unrooted) topology identical to the true gene trees. Results are averaged over all 24 datasets.

*RAxML trees yield high error rates*: The ML trees produced using the sequence-only method RAxML have high error rates, with the NRFD varying from a minimum of 0.06 (i.e. an error rate of 6%) on the datasets with sequence length 333 and mutation rates 1 and 3 (substitutions per site) (Supplementary Fig. S1, right-column), to a maximum of 0.17 on the datasets with sequence length 173 and a mutation rate of 10 (Supplementary Fig. S1, left-column). As expected, error rates are higher for datasets with high rates of sequence evolution as well as for those with shorter sequence length (Fig. 1). Biologists often collapse poorly supported branches in the inferred ML tree; however, we found that collapsing RAxML tree branches with low bootstrap support actually causes the error rate to increase (due to increased false negative rate in the unresolved trees). For example, at the commonly used threshold of 80%, the collapsed RAxML trees had an average NRFD of 0.14 compared with 0.097 for the uncollapsed RAxML trees.

*Methods that only model duplication-loss are ineffective*: Gene tree error-correction methods based on the duplication-loss model are ineffective at inferring gene trees affected by transfer, with average NRFD over all datasets of 0.088 (NOTUNG) and 0.079 (TreeFix). At low rates of DTL, NOTUNG and TreeFix do improve on the error rate of the RAxML trees by ∼30–40%, but for higher rates, they often performed even worse than RAxML.

*AnGST outperforms MowgliNNI*: Both AnGST and MowgliNNI improve significantly on the accuracy of the RAxML trees, with AnGST outperforming MowgliNNI on 23 of the 24 datasets. This is in spite of the fact that MowgliNNI was run on simulated species trees with perfect dating information while AnGST ignored all dating information. The average NRFD over all datasets are 0.032 (AnGST) and 0.039 (MowgliNNI), suggesting that, in general, AnGST is a more effective method than MowgliNNI.

*TreeFix-DTL infers the most accurate gene trees*: TreeFix-DTL offers consistent, high-quality performance, outperforming all other methods under most conditions and decreasing the error rate of RAxML trees by an average of 71.1%. The NRFD of the TreeFix-DTL trees varies from 0.006 (on the datasets with sequence length 333 and mutation rates 1 and 3) to 0.074 (on the datasets with sequence length 173 and a mutation rate of 10), and the average NRFD over all datasets is 0.028. Furthermore, TreeFix-DTL produced gene tree topologies completely identical to the true gene trees (i.e. with an NRFD of zero) 38.2% of the time (averaged over all datasets), compared with 3.0% for RAxML and 29.1% for AnGST.

In summary, even though our simulation study does not model the many idiosyncracies of real sequence evolution, such as insertions and deletions, lineage specific rate variation, etc., our analysis demonstrates the limitations of RAxML, and of methods developed for gene tree error correction under the duplication-loss model, at accurately inferring gene trees affected by transfer. We found a great improvement in accuracy by using gene tree error-correction methods designed for handling horizontal transfer, and TreeFix-DTL is the most accurate method overall, producing gene trees that have, on average 71.1% fewer topological errors than RAxML and over 10-fold higher rates of inferring the gene tree topology perfectly. TreeFix-DTL also has 12.5% fewer topological errors and 31.8% higher rates of inferring perfect gene tree topologies than AnGST. Importantly, neither TreeFix-DTL nor AnGST require species trees to be dated, while MowgliNNI does; yet both TreeFix-DTL and AnGST significantly outperform MowgliNNI even with perfect species tree dating.

#### 3.1.1 Scalability and speed

We evaluated the performance of TreeFix-DTL on larger 100- and 200-taxon datasets and observed that the error rates were generally similar to those observed on the corresponding 50-taxon datasets (Supplementary Section S4, Supplementary Fig. S2). This suggests that TreeFix-DTL performance does not deteriorate as the number of taxa in the input trees increases. Furthermore, irrespective of the size of the dataset, TreeFix-DTL requires only about three times the time required to build the RAxML trees themselves (Supplementary Table S3).

#### 3.1.2 Robustness to event costs

To use the DTL-reconciliation model, one must assign costs for the different evolutionary events. Based on existing literature (David and Alm, 2011), we chose costs of 2, 3 and 1 for DTL, respectively. To assess the impact of using different costs on gene tree inference, we ran TreeFix-DTL on the 50-taxon datasets from the basic simulation setup with all three event costs set to 1 (the simplest and most unbiased setting of event costs possible) and observed that performance was only slightly affected by using these alternative event costs (Supplementary Fig. S3). This demonstrates the robustness of TreeFix-DTL to the actual event costs used for DTL-reconciliation. We expect MowgliNNI and AnGST to be similarly robust to changes in event cost assignments.

#### 3.1.3 Robustness to very high rates of DTL, and other parameters

To study the performance of the methods on datasets with very high rates of DTL, we created gene trees with very high rates of DTL on the 50-taxon species trees from the basic simulation setup (see Supplementary Table S1). We refer to the resulting gene trees as *veryHigh-DTL* gene trees. These gene trees contained, on average, 109 leaf nodes, 10.0 duplications, 20.6 transfers and 6.9 losses (Supplementary Table S2). We created these datasets for mutation rates 1 and 5, and sequence length 333. Even on these veryHigh-DTL gene trees, TreeFix-DTL was effective at inferring gene trees accurately (Supplementary Fig. S4C). Specifically, the average normalized RF distances for RAxML, AnGST and TreeFix-DTL on these datasets were 0.065, 0.047 and 0.040, respectively. Note, however, that the relative decrease in the accuracy of TreeFix-DTL and AnGST on this dataset also suggests that gene families with extremely high rates of DTL may not benefit from those methods. We also evaluated the performance of RAxML, AnGST and TreeFix-DTL on two additional datasets with different ratios of DTL, and shorter alignments and found that TreeFix-DTL performed remarkably well in both cases (Supplementary Section S5, Supplementary Fig. S4A and B).

#### 3.1.4 Robustness to inaccurate species tree topologies

Error-correction methods use a known species tree topology to error-correct gene trees. To assess the impact of species tree errors on inference accuracy, we ran TreeFix-DTL and AnGST on the 50-taxon gene trees from the basic experimental setup with species trees that had been corrupted with one and three random NNI operations. (MowgliNNI could not be evaluated since it requires a dated species tree.) We found that both TreeFix-DTL and AnGST are robust to minor species tree errors and still substantially improve on RAxML gene trees despite these species tree topological inaccuracies (Supplementary Table S4). For example, for the datasets in which the species trees had one [three] NNI error(s), the average error rate of the TreeFix-DTL and AnGST trees were 61.3 [40.0%] and 59.1% [38.6%] smaller than that of the RAxML trees, respectively.

### 3.2 Impact on recovering duplications, transfers, and losses

We now study the impact of using more accurate gene trees on the ability to correctly recover DTL events. For the gene trees from the 50-taxon simulated datasets, inferred using RAxML, AnGST, and TreeFix-DTL, we applied DTL-reconciliation using RANGER-DTL (Bansal *et al.*, 2012) with default parameters. For MowgliNNI gene trees, we used the reconciliation returned by MowgliNNI itself (which makes use of the dating information on the species tree to improve accuracy). We then compared the resulting reconciliations to the true evolutionary histories, looking at both estimated event counts (Supplementary Fig. S5) and the accuracy of proposed events (Fig. 2). Note that we used the strictest definition of an event in our analysis; i.e. for a proposed event to be correct, it must be inferred at the correct location in both the gene tree and species tree; see Supplementary Section S6 for further details on how events were defined and compared.

For events based on inferred gene trees, we found that those based on RAxML trees are misleading and those based on TreeFix-DTL trees are much more accurate. In particular, RAxML trees greatly overestimate transfers and losses, leading to roughly twice as many transfers and losses as TreeFix-DTL and the actual implanted counts; this is also reflected in the very low precision of RAxML + RANGER-DTL at recovering transfers and losses. When compared with RAxML trees, DTL-reconciliation on TreeFix-DTL trees shows better sensitivity at recovering duplications (89.7 versus 80.3%), transfers (70.9 versus 65.6%) and losses (86.5 versus 76.3%) and substantially better precision at recovering transfers (74.4 versus 36.9%) and losses (76.3 versus 31.0%) (precision of recovered duplications is comparable at 80.6 versus 79.8%). By comparing the counts estimated on the TreeFix-DTL trees to the true counts, we observed that TreeFix-DTL has a tendency to slightly underestimate the number of transfers at the cost of slightly overestimating the number of duplications and losses; however, TreeFix-DTL shows the best overall performance at recovering transfers and losses.

Our experiments also show that DTL-reconciliation is highly accurate at recovering duplications, transfers, and losses on the true gene trees (Fig. 2). Specifically, applying DTL-reconciliation on the true gene tree topologies yielded an average sensitivity and precision, respectively, of 97.9 and 93.8% for duplications, 91.6 and 95.6% for transfers and 91.9 and 84.6% for losses (Fig. 2). Amazingly, DTL-reconciliation continues to be highly accurate even when gene trees have very high rates of DTL. For instance, when we applied DTL-reconciliation to the true gene trees from the very High-DTL datasets, we observed sensitivity and precision of 95.3 and 94.1% for duplications, 81.3 and 85.1% for transfers and 76.0 and 70.5% for losses.

### 3.3 Resolving gene tree-species tree conflict in cyanobacteria

To demonstrate the implications of differences in gene tree inference on a real dataset, we applied TreeFix-DTL to a set of 1128 protein-coding gene families from 11 completely sequenced cyanobacterial genomes (Zhaxybayeva *et al.*, 2006). Each of these gene families is single-copy and represents a gene shared by at least 9 of the 11 cyanobacterial genomes. Study of such single-copy, presumably orthologous, genes families play an important role in determining species phylogenies, but analyses have revealed many competing results (see, e.g. Bapteste *et al.*, 2004; Lerat *et al.*, 2003). However, previous inferences on cyanobacterial evolution have relied on gene trees reconstructed through NJ; therefore, here, we assess the extent to which model choice and gene tree inference affects downstream biological inferences. Given that all the gene trees in this dataset are single copy, we make the standard assumption that any gene tree-species tree incongruence is a result of (additive) horizontal gene transfer (and compensating losses) and that duplications do not occur. For brevity, we report only our findings in the main manuscript; details can be found in Supplementary Section S7.

We use TreeFix-TL and RANGER-TL to denote the variations of TreeFix-DTL and RANGER-DTL in which duplications are not modeled; i.e. the duplication cost is set to infinity. As with the simulated datasets, we found that accounting for possible gene tree error using TreeFix-TL greatly reduces the number of estimated transfers and losses. In particular, while many NJ and ML gene trees (the latter inferred using RAxML under the PROTGAMMAJTT model) were incongruent to the species phylogeny (NJ: 16 trees with NRFD 0, mean distance = 0.430; RAxML: 16 trees with NRFD = 0, mean distance = 0.423) and thus imply many transfer events, TreeFix-TL removed nearly all incongruence (856 trees with NRFD = 0, mean distance = 0.065), resulting in far fewer events (using RANGER-TL, 301 transfers, 573 losses, a reduction of 88.6 and 67.4%, respectively, compared with RAxML). Using TreeFix-DTL rather than TreeFix-TL resulted in gene trees with higher incongruence (712 trees with NRFD = 0, mean distance = 0.100), though TreeFix-DTL and TreeFix-TL trees were mostly similar (880 or 78.0% of gene trees were congruent between the program settings, NRFD = 0.058). Altogether, these results suggest that, for many gene families, there is insufficient phylogenetic signal to either support or reject a single gene tree topology. Similarly, there is insufficient signal to support or reject the majority of transfer events estimated on NJ and RAxML gene trees. At the same time, our analysis also identifies ∼300 well-supported transfer events.

**Fig. 3.**
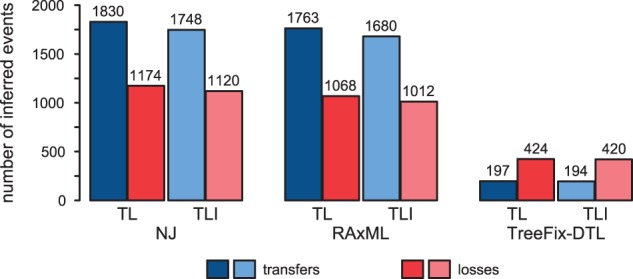
Cyanobacterial gene family evolution. Event counts are aggregated over 769 cyanobacterial gene families for which the reconciliation model of Stolzer *et al.* (2012) did not infer temporally infeasible or conflicting multiple optimal solutions. Applying TreeFix-TL to the gene families drastically reduces the number of estimated events

Next, we study the effect of model choice and the impact of possible gene tree error on inferences of temporal infeasibility, conflicting optimal solutions and ILS in cyanobacteria. Our analysis is motivated by the recent work of Stolzer *et al.* (2012), in which the authors proposed a reconciliation model for explaining gene tree-species tree incongruence through DTL and ILS, then demonstrated its impact through an empirical study on cyanobacteria. Here, we revisit two of their observations: (i) temporally infeasible and conflicting multiple optimal solutions are prevalent in real data and (ii) ILS can lead to overestimation of other events. Using NOTUNG (Stolzer *et al.*, 2012), we repeated their analysis using only transfer loss (TL) and transfer loss-ILS models of reconciliation (with default event costs) applied to TreeFix-TL gene trees, and we find that temporal infeasibility and conflicting multiple optimal solutions affect only 0.6% and 0–0.4% of gene trees, far fewer than the ∼10 and ∼20% previously reported. Furthermore, if we consider different gene trees and reconciliation models and use the same filtering criteria as Stolzer *et al.* (2012), we find that accounting for possible gene tree error reduces the impact of ILS-aware reconciliation (Fig. 3, Supplementary Table S5). Our results suggest that gene tree error correction can greatly impact biological inferences.

## 4 Discussion

In this study, we have shed light on the complications of gene tree inference in the presence of horizontal gene transfer and demonstrated that our new method, TreeFix-DTL, outperforms all existing methods at accurately inferring gene trees with transfer.

TreeFix-DTL builds more accurate gene trees by addressing the problem of phylogenetic uncertainty. Several complementary approaches exist to help build more accurate gene trees. These include methods for constructing more accurate sequence alignments (e.g. Liu *et al.*, 2009), more accurate substitution models that can better model the evolutionary process (e.g. Whelan *et al.*, 2011), etc. Treefix-DTL can be used in conjunction with these approaches to further improve gene tree accuracy.

Our results show that, even after error correction with TreeFix-DTL or other methods, the inferred gene trees are often not perfectly correct (Table 1). This is due to the fact that perfect inference requires that each clade in the gene tree be inferred correctly. Thus, even though gene tree error correction greatly improves the accuracy of the gene trees, we caution against assuming that the inferred gene trees represent fully accurate evolutionary histories.

In our analysis, we observed that AnGST performed quite well on our test datasets, delivering consistently good results and even surpassing the accuracy TreeFix-DTL in some cases. Thus, the simple approach used by AnGST may be worth studying and refining further and could be especially useful when rapid error correction is desired. One of the difficulties with using AnGST is that users must decide on the number of bootstrap replicates to use as input for AnGST. The number of bootstrap replicates used has a direct impact on the accuracy of the inferred gene tree; using too few bootstraps reduces the search space, but using too many leads to over-fitting.

The accuracy and scalability of TreeFix-DTL can be further improved by making the local search step more efficient. More efficient search techniques or faster algorithms for local search would make it possible to efficiently handle larger input instances and to further improve gene tree accuracy. Currently, if TreeFix-DTL encounters multiple gene trees with statistically equivalent likelihood and with the same minimum reconciliation cost, it outputs the gene tree with the highest likelihood score. In the future, it might be instructive to consider all gene trees with statistically equivalent likelihood scores and minimum reconciliation costs, and to study the similarities and differences in the alternative gene tree topologies.

TreeFix-DTL assumes that among all statistically equivalent candidates for the gene tree, the one with minimum reconciliation cost is most likely to be the true tree. However, the true evolutionary history of a gene family need not always be most parsimonious. It may therefore help to relax this assumption and to develop such an approach further, possibly by incorporating probabilistic models of DTL-reconciliation (e.g. Tofigh, 2009) that would allow for integrating over all possible reconciliations for a given gene tree.

Finally, in this study, we have focused specifically on gene trees affected by duplications, transfers, and losses and do not explicitly model ILS. Therefore, TreeFix-DTL may remove weak, but biologically meaningful, signals of deep coalescence since such events are intrinsically orthogonal to the species tree under the DTL-model. This could partially account for our finding of low ILS rates within cyanobacteria.

## Funding

This work was supported by a National Science Foundation [CAREER award 0644282 to M.K.], National Institutes of Health [RC2 HG005639 to M.K.], National Science Foundation AToL [0936234 to E.J.A. and M.K.], and startup funds from the University of Connecticut [to M.S.B.].

*Conflict of Interest*: none declared.

## Supplementary Material

Supplementary Data
