# Supplementary material for: Improved gene tree error correction in the presence of horizontal gene transfer
Source: Bioinformatics. 2014 Dec 5;31(8):1211–8. doi: 10.1093/bioinformatics/btu806 (PMC4393519; doi:10.1093/bioinformatics/btu806)
Supplement: Supplementary Data [file supp_31_8_1211__index.html]

Improved gene tree error correction in the presence of horizontal gene transfer — Supplementary Data 

# Improved gene tree error correction in the presence of horizontal gene transfer

## Supplementary Data

files

**Files in this Data Supplement:**

- Supplementary Data - pdf file
