# Supplementary material for: Improved gene tree error correction in the presence of horizontal gene transfer
Source: Bioinformatics. 2014 Dec 5;31(8):1211–8. doi: 10.1093/bioinformatics/btu806 (PMC4393519; doi:10.1093/bioinformatics/btu806)
Supplement: Supplementary Data [file supp_btu806_btu806-Supplement.pdf]

Improved Gene Tree Error Correction in the Presence of Horizontal Gene Transfer – Supplementary Information

Mukul S. Bansal, Yi-Chieh Wu, Eric J. Alm, Manolis Kellis

S.1 TreeFix-DTL Pseudocode

For any gene tree T, let $c(T;S)$ denote the reconciliation cost of T and S, $\delta(T;T_{ML};A)$ denote the difference in likelihood between T_{ML} and T, and $p(T;T_{ML};A)$ denote the p-value for the test statistic $\delta(T;T_{ML};A)$ as computed by the SH-test. In the pseudocode below, T^* and c^* keep track of the best tree and lowest reconciliation cost observed so far during the search, and δ^* keeps track of the difference in the likelihoods of the input ML gene tree and the tree T^*.

1. Let $c^* = c(T_{ML};S)$, and initialize $T^* = T_{ML}$ and $\delta^* = 0$.
2. Make a fixed number of proposals (100 in the current implementation) for the gene tree topology, and denote this collection of proposals by X. Compute $c(T;S)$ for each $T \in X$.
3. Sort the trees in X by increasing cost, and retain only those trees for which $c(T;S) \leq c^*$. Denote this new (sorted) collection of trees by Y.
4. For each $T \in Y$, compute $p(T;T_{ML};A)$ and $\delta(T;T_{ML};A)$. If $p(T;T_{ML};A) \geq \alpha$ and either (i) $c(T;S) < c^*$ or (ii) $c(T;S) = c^*$ and $\delta(T;T_{ML};A) < \delta^*$, set $T^* = T$, $c^* = c(T;S)$, and $\delta^* = \delta(T;T_{ML};A)$, and go to the next step. Else consider the next proposal.
5. Repeat Steps 2–4 for a predetermined number of iterations (default 1000), or until $c^* = 0$.
6. Output T^*.

Note that the actual implementation of TreeFix-DTL generates the proposals and implements the local search step in a particular way to avoid getting caught in local minima. Specifically, during each search iteration, we start with the current gene tree and propose a new tree by performing a random NNI or SPR rearrangement; this proposal is always accepted if it is statistically equivalent to the input gene tree and has lower reconciliation cost than the current optimal gene tree, and accepted with some predefined probability otherwise. The next proposal is then generated based on the, potentially new, current gene tree.

Complexity analysis. Let s denote the number of search iterations performed by the algorithm. Each of these iterations involves evaluating a constant number (100 in the current implementation) of tree topologies. The time spent on each tree is dominated by two additive components. First, the time complexity of performing the SH-test and of computing the likelihood for a given tree topology. And second, the time required to perform DTL-reconciliation. Let m and n denote the sizes of the gene tree and species tree, respectively, and let a denote the alignment length. In our method, the calculations for the first component are performed heuristically by RAxML. We denote this cost as $r(a,m)$, since it depends on the alignment

length and on the size of the input gene tree. The second component has a time complexity of $O(mn)$. The total time complexity of our algorithm is thus $O(s(r(a,m)+mn))$.

S.2 Choice of Parameters for Simulated Datasets

The low-DTL, medium-DTL and high-DTL gene trees had, on average, 52.3, 70.4, and 91.3 leaf nodes, 1.2, 2.8, and 5.0 duplications, 2.2, 5.5, and 9.9 transfers, and 2.1, 2.3, and 2.9 losses, respectively (Table S2). To generate these datasets using the probabilistic model of gene duplication, transfer, and loss (Tofigh, 2009; Tofigh *et al.*, 2011), we used the duplication, transfer, and loss rates given in Table S1. These rates represent the probability of a particular event type occurring per gene lineage per unit branch length on the species tree, and our 50-taxon species trees had an average branch length of 11.24 per tree (and 0.115 per edge). The ratio of duplications, transfers, and losses that we used was based on our analysis of a 4736 gene tree, 100 species dataset from David and Alm (2011), which consists of predominantly prokaryotic species sampled broadly from across the tree of life. In general, we chose the duplication, transfer, and loss rates for which the reconstructed RAxML trees yielded duplication, transfer, and loss counts in a ratio roughly similar to those observed on that dataset. Specifically, the reconstructed RAxML trees for our low-DTL, medium-DTL, and high-DTL datasets showed, on average, 1.25, 2.91, and 4.89 duplications, 5.88, 10.04, and 15.49 transfers, and 4.88, 5.86, and 7.10 losses, respectively (see also the actual number of implanted events as shown in Table S2). Likewise, the choice of 173 for the amino acid alignment length is based on an analysis of the same biological dataset, where we observed that the median alignment length was 173. The choice of the other alignment length, 333, is motivated by the fact that the typical prokaryotic gene length is approximately 1000 base pairs (Koonin and Wolf, 2008). Our choice of mutation rates is meant to span the range from slow evolving (or less diverged) gene families to fast evolving (or highly diverged) gene families. The average branch lengths for our low-DTL datasets, with mutation rates 1, 3, 5, and 10, were 0.11, 0.33, 0.55, and 1.1, respectively (in terms of average number of mutations per site). The corresponding mutation rates for the medium-DTL datasets were 0.10, 0.3, 0.5, and 1.0, respectively, and for the high-DTL datasets they were 0.095, 0.29, 0.48, and 0.95, respectively. The average branch length in the biological dataset was 0.264.

S.3 Choice of Parameters for RAxML, NOTUNG, TreeFix, MowgliNNI, and AnGST

For reconstructing gene trees using RAxML we used the following command:

```
raxmlHPC -f a -x 12345 -p 12345 -s input.fasta -m PROTGAMMAJTT -#100
```

NOTUNG and MowgliNNI require the input gene tree to be labeled with bootstrap supports at each edge. We inferred these bootstrap support values based on the 100 rapid bootstraps from the RAxML runs. Both NOTUNG and MowgliNNI also require a bootstrap cutoff percentage which specifies the edges in the gene tree can be modified; for NOTUNG we used the default value of 90% of the maximum bootstrap, and for MowgliNNI we used a cutoff of 80% based on the author recommendation from Nguyen *et al.* (2012). In addition, MowgliNNI requires that the input gene tree be rooted and that the species tree be fully dated. Our simulated species trees were fully dated and were used as input for MowgliNNI, and we ran MowgliNNI on all possible rootings of the input gene tree. AnGST requires as input a set of bootstrap replicates for the gene tree. Since, AnGST does not specify any default settings for the number of bootstrapped gene trees to use, we tried two settings: 10 bootstraps as previously used by the authors of AnGST, and a more natural value of 100 bootstraps more likely to be used by most users. We discovered that performance was significantly improved by using 100 bootstraps instead of 10 (results not shown). Thus, we used the 100 rapid bootstraps

from RAxML as input to AnGST. TreeFix was run using a thorough search setting ("long" version as defined by Wu *et al.* (2013)), that corresponds to the (default) search setting used for TreeFix-DTL.

S.4 Scalability and Speed

To study the scalability and performance of the methods on larger datasets (with more taxa), we created datasets with 100- and 200-taxon species trees using the same methodology we used to create the 50-taxon datasets. We created these larger datasets for medium-DTL, mutation rates 1 and 5, and sequence length 333. The gene trees for the 100-taxon datasets had, on average, 144.3 leaf nodes, 5.3 duplications, 11.3 transfers, and 4.3 losses, while the 200-taxon datasets had, on average, 290.7 leaf nodes, 10.1 duplications, 21.3 transfers, and 9.1 losses per gene tree. We observed that the error rates of the TreeFix-DTL trees on these 100- and 200-taxon datasets are generally similar to those observed on the corresponding 50-taxon datasets (Supplementary Figure S2). This suggests that the performance of the method does not deteriorate as the number of taxa in the input trees increases. Furthermore, TreeFix-DTL is fast enough to be easily applied to gene trees and species trees with hundreds of leaves (Supplementary Table S1). For instance, on the 100- and 200-taxon datasets, TreeFix-DTL required 13.8 and 43.5 hours per tree, respectively, on average (including the time to build the initial RAxML tree), when executed on a compute cluster with each node consisting of an 800 MHz AMD Opteron processor and 4 GB of RAM. This compares favorably to the 4.1 and 15.0 hours of average runtime required to build just the initial RAxML trees themselves (using the thorough search settings as previously described). Thus, gene tree inference using TreeFix-DTL is only about three times as slow as doing a thorough sequence-only reconstruction using RAxML. AnGST is very efficient in general (assuming the bootstrap trees have already been computed), especially for the smaller (50- and 100-taxon) input instances; but, due to excessive memory requirements, we found it hard to run AnGST on datasets with more than 200 taxa (on a computer with 4 GB of RAM) (Table S1). Runtimes for MowgliNNI were roughly twice as high as those for TreeFix-DTL.

TreeFix-DTL relies on a local search strategy to find more accurate gene trees, and its performance depends on the number of local search steps allowed during the search. By default, TreeFix-DTL executes 1000 local search steps per run, providing a good trade-off between running time and accuracy for a wide range of tree sizes. To study the impact of using more local search steps, we also ran TreeFix-DTL with 5000 local search steps per run on the 100- and 200-taxon datasets (results not shown). As expected, we observed that accuracy improved with the use of more local search steps, with the more exhaustive version having, on average, a 15% smaller error-rate than the default parameterization of TreeFix-DTL. This additional increase in accuracy, however, comes at the cost of a five fold increase in running time. Nonetheless, the number of local search steps can be increased to obtain even better accuracy whenever accuracy is paramount, or when the number of leaves in the gene trees or species trees involved exceeds a few hundred.

S.5 Additional Experiments

Robustness to duplication, transfer, and loss ratios. Since the datasets considered in the basic experimental setup all have more transfers than duplications (as learnt from the biological dataset from David and Alm (2011), we also tested the performance of AnGST and TreeFix-DTL on datasets in which there were more duplications than transfers. The idea is to test if the performance of TreeFix-DTL depends on the ratio of events in the dataset. Specifically, we created new gene trees that had, on average, 5.2 duplications, 4.7 transfers, and 3.9 losses. We created these datasets for mutation rates 1 and 5, and sequence length 333. We observed that TreeFix-DTL is just as effective at inferring gene trees on these datasets as in our previous experiments, decreasing the error rate of the RAxML trees by 70.5% (Supplementary Figure S4A).

Performance on short genes (alignment length 75 amino acids). We observed that over 10% of the 4736 gene trees in the biological dataset from David and Alm (2011) had a multiple sequence alignment length of less than 75 amino acids. Thus, to test the ability of the methods to infer accurate gene trees on short alignments, we created datasets, using the 50-taxon species trees and the same basic experimental setup described earlier but with sequence length 75. We created these datasets for medium-DTL and mutation rates 1 and 5. On these datasets with very short alignments, the error rates of the inferred gene trees were, unsurprisingly, substantially higher than for gene trees inferred using the length 173 or 333 alignments (Supplementary Figure S4B). For instance, for the dataset with mutation rate 1, RAxML, AnGST, and TreeFix-DTL had NRFD of 0.185, 0.075, and 0.064, respectively. In spite of these higher absolute error rates, the average error rate for the TreeFix-DTL trees (8.2%) is much smaller than the average error rate for the RAxML trees (21.2%).

S.6 Event Recovery

We used the strictest definition of an event in our analysis; that is, for a proposed event to be correct, it must be inferred at the correct location in both the gene tree and species tree. In particular, we require that

- for duplications, the same gene duplicates in the same species. That is, two duplications are identical if the duplication nodes yield the same children subtrees and the duplication nodes are mapped to the same species.
- for transfers, the same gene transfers from the same species. That is, two transfers are identical if the transfer nodes yield the same children subtrees, the transfer edges are the same, and the transfer node is mapped to the same (donor) species. Note that the recipient species is not tracked because our simulator did not retain this information.
- For losses, the same gene is lost in the same species. That is, two losses are identical if they occur along the same branch of the gene tree (this branch is required to exist, else the proposed loss is incorrect), and the losses occur in the same species.

For each gene tree, we compared the estimated events to the true events (known through simulation), applying the above definitions to classify each estimated event as correct or incorrect. For each event type (duplication, transfer, loss), we then divided the number of correct events (of that type) by either the total number of true events (to determine sensitivity) or by the total number of estimated events (to determine precision).

We point out that, when multiple optimal DTL reconciliations existed, event inference accuracy was based on a single solution selected at random from the set of multiple optimal solutions. Also, since RANGER-DTL seeks optimal, but not necessarily time-consistent, DTL reconciliations, it is possible that some of the recovered reconciliations are time-inconsistent. However, note that neither random optima nor time-inconsistency are expected to bias our results one way or another since our results are averaged over 2400 50-taxon gene tree/species tree pairs.

Finally, we note that applications exist for which relaxed event definitions may be more appropriate. For example, to differentiate between orthologs, paralogs, and xenologs, we are only interested in the gene tree topology and the location of (respectively, speciation, duplication, and transfer) events in the gene tree. That is, the species mappings can be ignored.

S.7 Analysis of Cyanobacterial Gene Families

To analyze the cyanobacteria gene families, we obtained a species tree, multiple sequence alignments, and PHYLIP (Felsenstein, 1989) NJ gene trees from Zhaxybayeva *et al.* (2006). We then used RAxML and TreeFix-TL to infer gene trees. Finally, we applied RANGER-DTL to the NJ, RAxML, and TreeFix-DTL gene trees to estimate events (and also to reroot the gene trees for the unrooted NJ and RAxML trees). For all programs, we used the same parameters as in our simulation study.

Next, we considered the analysis of Stolzer *et al.* (2012), which considered Transfer-Loss (TL) and Transfer-Loss-ILS (TLI) reconciliation models, as well as Duplication-Transfer-Loss (DTL) and Duplication-Transfer-Loss-ILS (DTLI) models, and filtered gene families by removing any families that contained either temporally infeasible or conflicting multiple optimal solutions. Using this process, they obtained a set of 314 families in which they compared estimated event counts. By considering only TL and TLI models and using the same filtering criteria, we obtain a set of 769 gene families, or 2.45× that considered by Stolzer *et al.* (2012). With this larger set of families, applying the TLI reconciliation model to the original NJ trees decreased the number of estimated transfers and losses by only 4.48% and 4.60%, respectively, over the TL model (Figure S3); this is a far smaller reduction than that reported by Stolzer *et al.* (2012) (15–18% decrease in duplications + transfers, up to 20% decrease in losses), though we note that the larger differences remain if we relax the filtering criteria (Table S5). With the more accurate TreeFix-DTL gene trees, the similarity between estimated events using the two models is even more dramatic, with the TLI reconciliation model decreasing the inferred number of transfers and losses by only 1.52% and 0.94%, respectively, over the TL model.

DTL rate	Duplication rate	Transfer rate	Loss rate
low-DTL	0.1	0.2	0.2
medium-DTL	0.2	0.4	0.2
high-DTL	0.3	0.6	0.2
veryHigh-DTL	0.6	1.2	0.6

Table S1: **Parameters used for simulated datasets.** The table shows the duplication, transfer, and loss rates used with the probabilistic model of gene tree evolution (Tofigh, 2009; Tofigh *et al.*, 2011) to generate the low-, medium-, high-, and veryHigh-DTL gene trees used in the simulation study.

DTL rate	Num. duplications	Num. transfers	Num. losses	Num. leaves
low-DTL	1.2	2.2	2.1	52.3
medium-DTL	2.8	5.5	2.3	70.4
high-DTL	5.0	9.9	2.9	91.3
veryHigh-DTL	10.0	20.6	6.9	109

Table S2: **Gene tree properties.** The table shows the average number of implanted duplications, transfers, and losses, and the average number of leaves in the low-, medium-, high-, and veryHigh-DTL gene trees.

Dataset type	Program	Runtime
50-taxon, Low DTL, Sequence length 173	RAxML	0.8 hr
	RAxML + AnGST	0.8 hr
	RAxML + TreeFix-DTL	2.7 hr
50-taxon, Medium DTL, Sequence length 173	RAxML	1.3 hr
	RAxML + AnGST	1.3 hr
	RAxML + TreeFix-DTL	4.85 hr
50-taxon, High DTL, Sequence length 173	RAxML	2.0 hr
	RAxML + AnGST	2.0 hr
	RAxML + TreeFix-DTL	7.9 hr
50-taxon, Low DTL, Sequence length 333	RAxML	1.5 hr
	RAxML + AnGST	1.5 hr
	RAxML + TreeFix-DTL	3.6 hr
50-taxon, Medium DTL, Sequence length 333	RAxML	2.1 hr
	RAxML + AnGST	2.1 hr
	RAxML + TreeFix-DTL	5.8 hr
50-taxon, High DTL, Sequence length 333	RAxML	3.1 hr
	RAxML + AnGST	3.1 hr
	RAxML + TreeFix-DTL	10.1 hr
100-taxon datasets (Medium DTL, Sequence length 333)	RAxML	4.1 hr
	RAxML + AnGST	4.1 hr
	RAxML + TreeFix-DTL	13.8 hr
200-taxon datasets (Medium DTL, Sequence length 333)	RAxML	15.0 hr
	RAxML + AnGST	16.0 hr
	RAxML + TreeFix-DTL	43.5 hr

Table S3: **Average runtimes for inferring gene trees.** The results for each category are averaged over all datasets generated for that category. RAxML was run with the command line option that produces 100 rapid bootstraps and executes the search heuristic 10 times. AnGST was run with 100 boootstrap trees as input, and TreeFix-DTL was run using its default settings. RAxML and TreeFix-DTL were run on a compute cluster where each node had an 800 MHz AMD Opteron processor with 6 cores and 4 GB of RAM (each run used a single core) and the times reported are CPU time. AnGST was run on a desktop computer with a 3.2 GHz Intel Core i3 processor and 4 GB of RAM and the times reported are wall time. Due to the different hardware used, runtimes for AnGST are not directly comparable to those of RAxML and TreeFix-DTL and are included only for completeness.

Program	Average error rate (NRFD)
RAxML	0.088
TreeFix-DTL on true species trees	0.027
AnGST on species trees with 1 NNI	0.036
AnGST on species trees with 3 NNIs	0.054
TreeFix-DTL on species trees with 1 NNI	0.034
TreeFix-DTL on species trees with 3 NNIs	0.053

Table S4: **Impact of species tree error.** Gene trees are inferred using topologically incorrect species trees (constructed through 1 NNI and 3 NNI operations). The results for each level of species tree error (number of NNI operations) is averaged over the four datasets (50-taxon, medium DTL, mutation rates 1 and 5, sequence lengths 333 and 173) with 100 input instances each.

Gene tree	Reconciliation	Trees	Infeasible	Conflicting	Transfers	Losses
NJ	TL	1022	94	12	2432	1663
	TLI	898	95	135	2051	1381
RAxML	TL	1029	80	19	2391	1515
	TLI	907	83	138	1985	1223
TreeFix-DTL	TL	1121	7	0	287	564
	TLI	1117	7	4	279	555

Table S5: **Event counts for cyanobacteria dataset.** For each gene tree method and reconciliation model, gene trees with temporally infeasible reconciliations or conflicting multiple optimal solutions are removed (independently for each set of programs; the number of conflicting families is counted only over the set of families with temporally feasible solutions). Event counts are then aggregated over the remaining set of gene families. Note that applying ILS-aware reconciliation to NJ trees greatly increases the percentage of conflicting or inconsistent trees (TL: 10.4%, TLI: 25.6%), with similar results for RAxML trees (TL: 9.6%, TLI: 24.4%), whereas applying ILS-aware reconciliation to TreeFix-DTL trees shows a much more modest difference (TL: 0.6%, TLI: 1.0%).

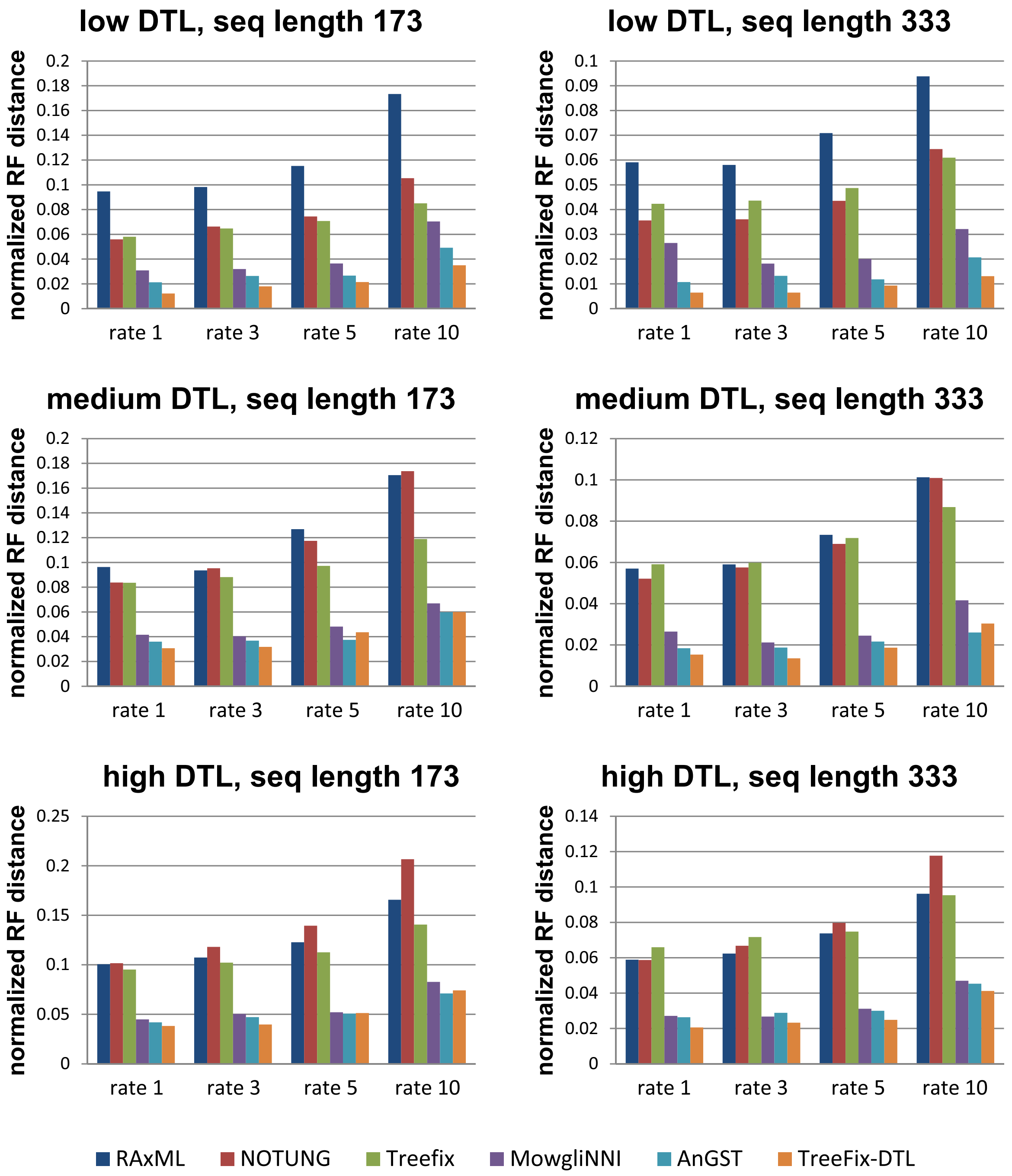

Figure S1: **Error rate of the different methods on simulated datasets of 50 taxa.** Error rates in terms of the NRFD are shown for gene trees inferred using RAxML, NOTUNG, TreeFix, MowgliNNI, AnGST, and TreeFix-DTL on the 24 simulated datasets. Each plot shows the results for a specific rate of duplication, transfer and loss (low-, medium-, or high-DTL), a specific sequence alignment length (173 or 333 amino acids), and for all four chosen rates of mutation (Rates 1, 3, 5, and 10).

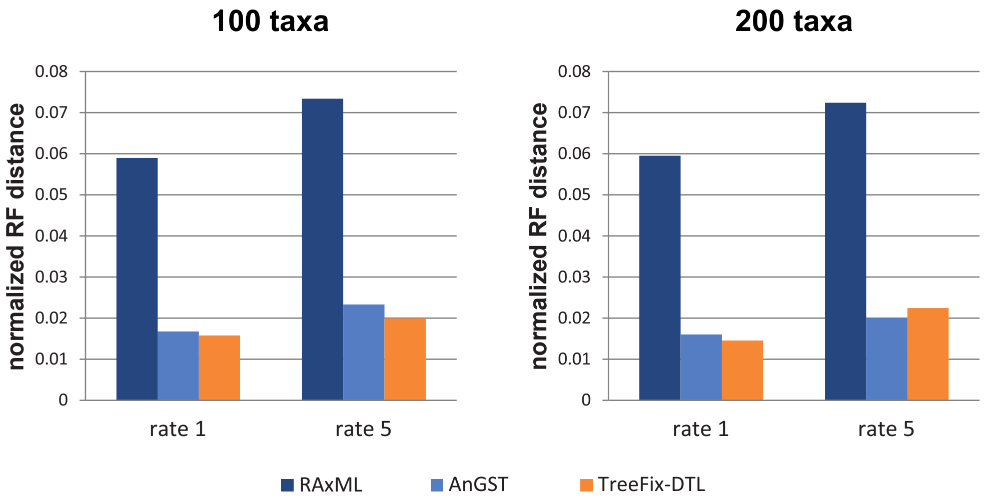

Figure S2: **Error rate on large simulated datasets of 100 and 200 taxa.** Error rates in terms of the NRFD for the two simulated 100-taxa datasets and the two simulated 200-taxa datasets.

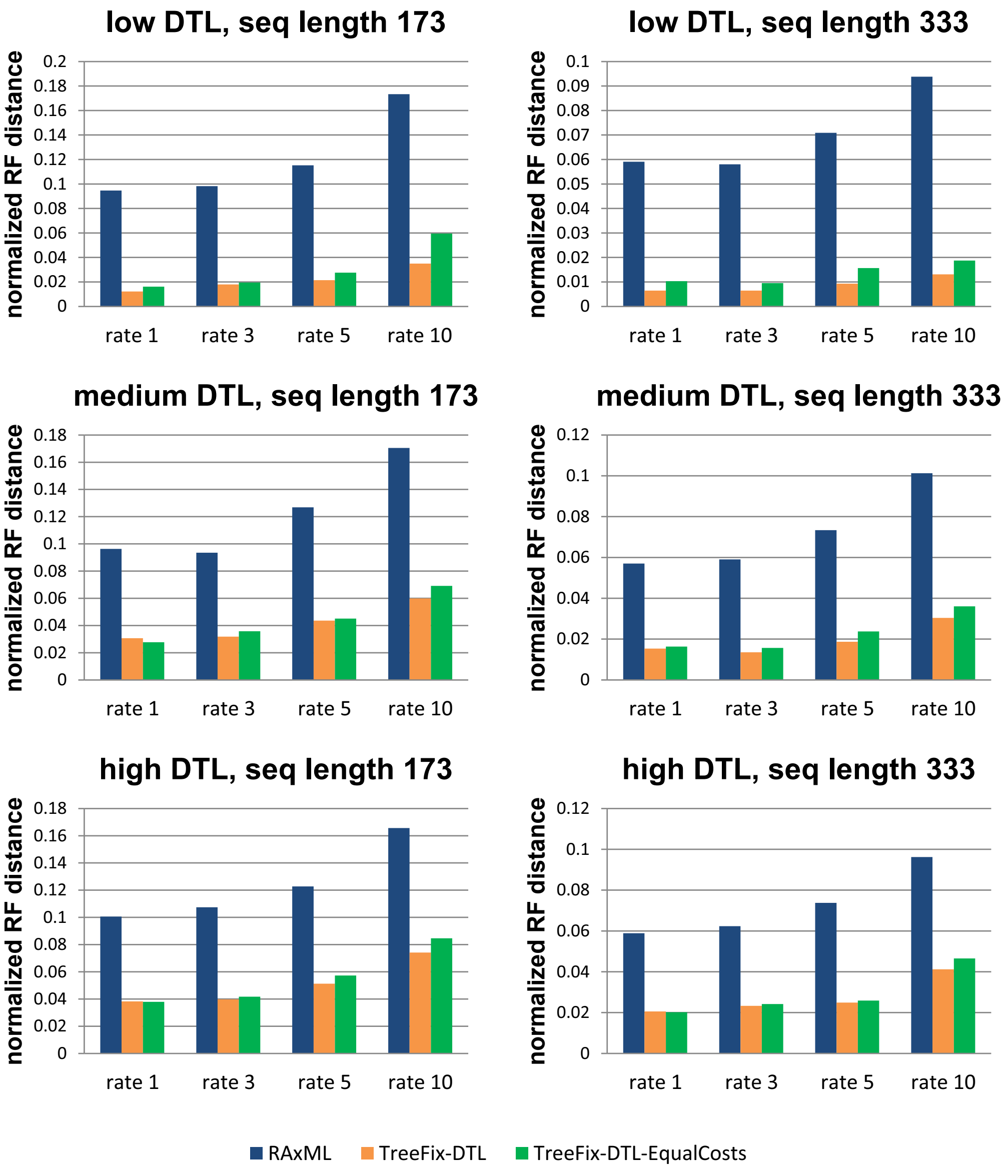

Figure S3: **Error rate using different event costs on simulated datasets of 50 taxa.** Error rates in terms of the NRFD are shown for 24 simulated datasets of 50 taxa. TreeFix-DTL-EqualCosts is the variant of TreeFix-DTL with costs for duplication, transfer, and loss events set to 1.

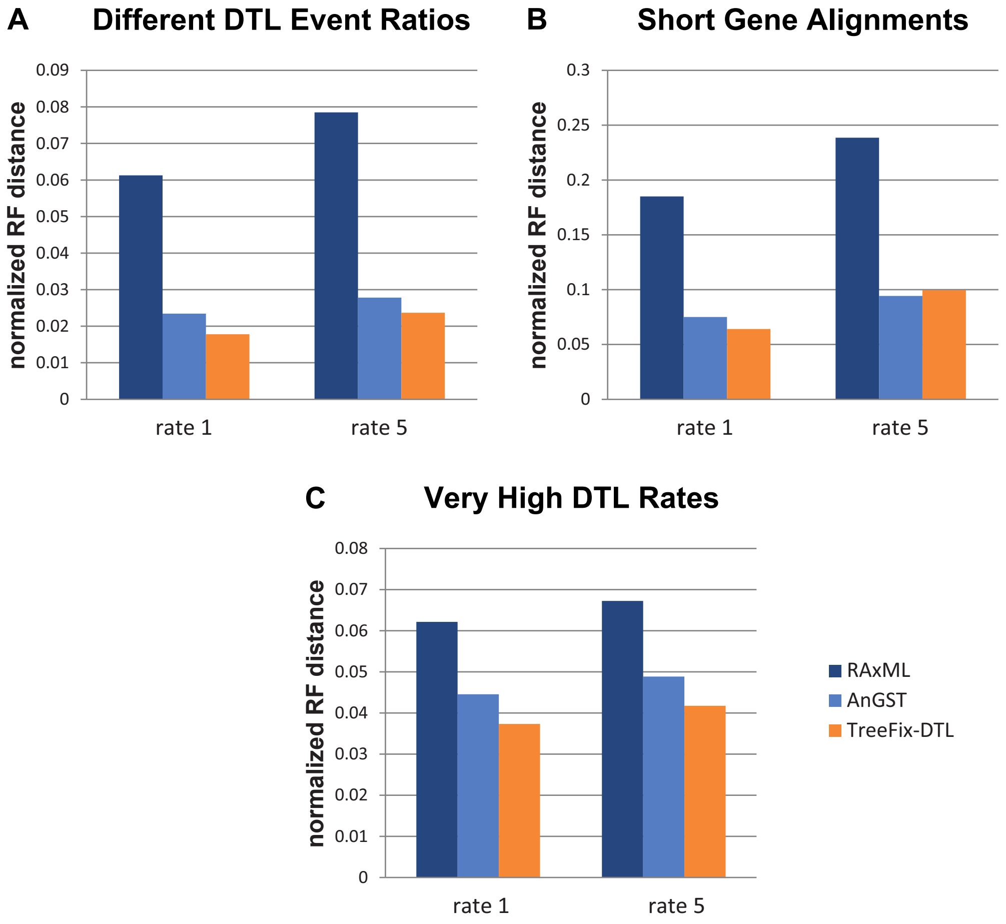

Figure S4: **Error rate on additional simulated datasets of 50 taxa.** Error rates in terms of the NRFD for the (A) two simulated datasets with a different ratio of duplication, transfer, and loss, (B) two simulated datasets with sequence length 75, and (C) two simulated datasets with very high rates of duplication, transfer, and loss.

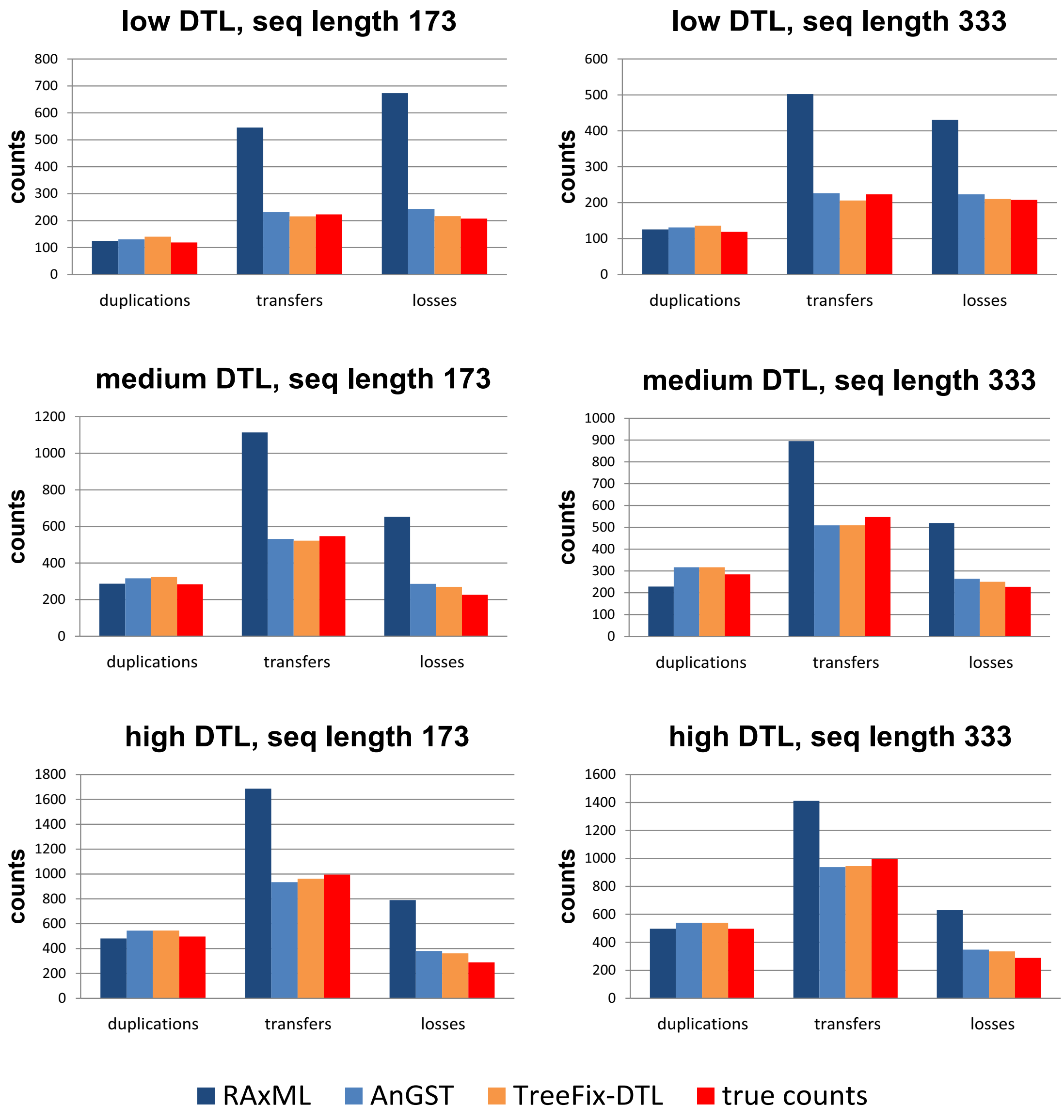

Figure S5: **Number of evolutionary events estimated for simulated datasets of 50 taxa.** Duplications, transfers, and losses are estimated using DTL-reconciliation. Each plot shows the results for a specific rate of duplication, transfer and loss (low-, medium-, or high-DTL), a specific sequence alignment length (173 or 333 amino acids), and averaged over the four chosen rates of mutation (Rates 1, 3, 5, and 10). The true (implanted) counts are also shown.

References

David, L. A. and Alm, E. J. (2011). Rapid evolutionary innovation during an archaean genetic expansion. *Nature*, **469**, 93–96.

Felsenstein, J. (1989). PHYLIP - phylogeny inference package (version 3.2). *Cladistics*, **5**, 164–166.

Koonin, E. V. and Wolf, Y. I. (2008). Genomics of bacteria and archaea: the emerging dynamic view of the prokaryotic world. *Nucleic Acids Res.*, **36**(21), 6688–6719.

Nguyen, T. H., Doyon, J.-P., Pointet, S., Chifolleau, A.-M. A., Ranwez, V., and Berry, V. (2012). Accounting for gene tree uncertainties improves gene trees and reconciliation inference. In B. J. Raphael and J. Tang, editors, *WABI*, volume 7534 of *Lecture Notes in Computer Science*, pages 123–134. Springer.

Stolzer, M., Lai, H., Xu, M., Sathaye, D., Vernot, B., and Durand, D. (2012). Inferring duplications, losses, transfers and incomplete lineage sorting with nonbinary species trees. *Bioinformatics*, **28**(18), 409–415.

Tofigh, A. (2009). *Using Trees to Capture Reticulate Evolution : Lateral Gene Transfers and Cancer Progression*. Ph.D. thesis, KTH Royal Institute of Technology.

Tofigh, A., Hallett, M. T., and Lagergren, J. (2011). Simultaneous identification of duplications and lateral gene transfers. *IEEE/ACM Trans. Comput. Biology Bioinform.*, **8**(2), 517–535.

Wu, Y.-C., Rasmussen, M. D., Bansal, M. S., and Kellis, M. (2013). Treefix: statistically informed gene tree error correction using species trees. *Systematic Biology*, **62**(1), 110–120.

Zhaxybayeva, O., Gogarten, J. P., Charlebois, R. L., Doolittle, W. F., and Papke, R. T. (2006). Phylogenetic analyses of cyanobacterial genomes: Quantification of horizontal gene transfer events. *Genome Research*, **16**(9), 1099–1108.
